# Impact of matrix metalloproteinase 9 rs3918242 genetic variant on lipid-lowering efficacy of simvastatin therapy in Chinese patients with coronary heart disease

**DOI:** 10.1186/s40360-017-0132-y

**Published:** 2017-04-08

**Authors:** Yuanyuan Xu, Yan Wang, Jixin Zhi, Lichun Qi, Tong Zhang, Xueqi Li

**Affiliations:** Department of Cardiology, The Fourth Affiliated Hospital of Harbin Medical University, 37 Yiyuan Rd, Nangang Region, Heilongjiang Harbin, 150001 China

**Keywords:** HMG-CoA reductase inhibitor, Genetic polymorphism, MMP9, Coronary heart disease

## Abstract

**Background:**

Genetic variation of matrix metalloproteinase 9 (MMP-9) gene polymorphism has been suggested to modulate coronary heart diseases (CHD), yet the underlying mechanisms are not well understood.

**Methods:**

We investigated the association of MMP9 rs3918242 single nucleotide polymorphism with inflammation and lipid-lowering efficacy after simvastatin treatment in Chinese patients with CHD. Fasting serum lipid profile and plasma inflammatory mediators were determined at baseline in 264 patients with CHD and 186 healthy control subjects, and after HMG-CoA reductase inhibitor simvastatin treatment (20 mg/day) for 12 weeks in CHD subjects.

**Results:**

We found that plasma MMP-9, TNF-α and IL-10 levels were significantly elevated in patients with CHD compared to control subjects before treatment. The plasma MMP9 in CHD patients carrying rs3918242 CC, CT and TT genotypes were comparable. Interestingly, CHD patients carrying TT genotype had significantly higher level of triglyceride (TG) and low-density lipoprotein cholesterol (LDL-C) than those carrying CC genotype (*P* <0.05). Simvastatin treatment significantly reduced LDL-C, TG and plasma inflammatory mediator levels in CHD patients. The reduction of LDL-C upon simvastatin therapy was significantly greater in patients carrying TT genotype than those carrying CC genotype (*P* <0.05).

**Conclusions:**

MMP9 rs3918242 TT genotype is associated with elevated serum TG and LDL-C, and enhanced LDL-C-lowering response upon simvastatin treatment in Chinese patients with CHD.

**Clinical trial registration:**

This study was retrospectively registered at Chinese Clinical Trial Registry (Registration number: ChiCTR-ROC-17010971) on March 23^rd^ 2017.

## Background

The matrix metalloproteinases (MMPs) are a family of zinc-dependent endopeptidases collectively capable of degrading essentially all components of extracellular matrix [1]. One of the MMP family member, MMP9, has been shown to involve in the pathogenesis of cardiovascular diseases [2, 3]. Furthermore, MMP9 SNP rs3918242 and circulating MMP9 level are associated with coronary heart disease (CHD) progression and MI, arterial stiffness and cardiovascular disease mortality in patients with CHD [4–7], while the mechanisms are not completely understood.

CHD is characterized by elevated circulating pro-inflammatory mediators including TNF-a and IL-6, and reduced anti-inflammatory mediators, such as IL-10 [8]. The imbalance of pro-inflammatory and anti-inflammatory mediators contributed to CHD progression [9]. Several lines of evidence suggest a potential link of plasma MMP9 activity to plasma TNF-a level, and a negative association with plasma IL-10 level in chronic inflammatory conditions [10]. However, whether MMP9 rs3918242 polymorphism is associated with altered plasma MMP9 level and plasma inflammatory mediators in the Chinese patients with CHD has not been characterized [11].

The clinical beneficial effects of statin therapy in reducing the risk of coronary events and mortality in patients with coronary artery disease are believed to be the result of its cholesterol-lowering and anti-inflammatory actions [12, 13]. Studies have shown that statin therapy reduces circulating MMP9 level in CHD patients and decreases MMP9 secretion in experimental conditions [14, 15]. It is still unclear if the decreased MMP9 level or activity upon statin therapy might merely correlate with or modulate its lipid-lowering efficacy in patients with CHD.

In this study, we examined the association of MMP9 rs3918242 polymorphism on MMP9 level, lipid profiles and inflammatory mediators in the blood in response to simvastatin therapy in Chinese patients with CHD.

## Methods

### Research participants

The research protocol was approved by the Ethics Committee of Harbin Medical University. We recruited 264 coronary heart diseases patients (112 females, 152 males; 55–78 years old), who underwent coronary angiography in the fourth clinical hospital of Harbin medical university. Written informed consent was obtained from all the participants with a full explanation of the study. Coronary arteriograms were interpreted by two or more independent, experienced cardiologists in a blinded manner. CHD was defined as over 50% stenosis in at least one major vessel. The inclusion criteria were: (1) diagnosed of CHD (2) strictly abstained from smoking, alcohol and caffeine during treatment. The exclusion criteria were (1) subjects who had taken or were currently taking HMG-CoA reductase inhibitors; (2) individuals with liver or kidney failure or subject to liver or kidney transplantation, diabetes mellitus, dropsical nephritis and diagnosed myocardial infarction; (3) subjects who had taken any other lipid-lowering medication within 2 months before the study were also excluded. The control population consisted of 186 healthy subjects (90 females, 96 males; 52–79 years old) who had routine health check at medical examination center.

### Blood collection and biochemical analysis

All subjects were asked to take low-fat diet and administered simvastatin 20 mg per oral ever day at bedtime. Fasting serum lipids were determined before and after 12 weeks of statin treatment. Total cholesterol (TC), triglyceride (TG), high-density lipoprotein cholesterol (HDL-C), low density lipoprotein cholesterol (LDL-C) and very low density lipoprotein cholesterol (VLDL-C) in plasma were measured by enzymatic methods using an auto-analyzer. Plasma glucose levels were measured using an automated glucose oxidase method described before. Serum MMP9 levels were measured by enzyme-linked immunosorbent assay (ELISA) using the monoclonal antibody against MMP9 [16]. The plasma TNF-α level was quantified using a sandwich ELISA kit (rat TNF-α/TNFSF1A, Duoset, R&D Systems, Abington, UK) and IL-10 level was assessed using the ChemiArray Rat Cytokine Antibody Array I kit (Chemicon-Millipore).

### Genotyping of human MMP-9 gene 1562C > T polymorphism

Genomic DNA was extracted from peripheral blood mononuclear cells (PBMCs). The MMP9 gene rs3918242 polymorphism was determined by polymerase chain reaction-restriction fragment length polymorphism (PCR-RFLP) based protocol [13]. Briefly, a 435 bp region in the promoter of MMP-9 gene, −1809 to −1374 upstream of MMP-9 transcription start site (TSS), was amplified. Forward primer 5′-GCCTGGCACATAGTAGGCCC-3′, reverse primer 5′-CTTCCTAGCCAGCCGGCATC-3′. The amplification reaction mix included 20 ng genomic DNA, dNTP (0.8uM), forward and reverse primer (0.5uM each), Prozyme DNA polymerase (0.5 unit) in the PCR buffer. PCR reaction was performed according to manufacturer’s manual. Aliquots of the PCR product were then fragmented using SphI restriction enzyme, and the the digestion products were analyzed by 2% agarose gel electrophoresis.

### Statistical analysis

All statistical analyses were performed with SPSS software (version 13.0; SPSS, Chicago, IL, USA). Allele frequencies were estimated by gene counting. ANOVA was applied to compare average values of biochemical parameters between genotypes; Chi-square test was used to compare genotype and gene frequencies between the groups. Comparison between control and CAD subjects were performed using student’s *T* test. Comparison of parameters before and after statin treatment were conducted using paired student’s *T* test. All data were presented as the mean ± SD. A two-tailed *P*-value < 0.05 was considered as statistically significant for all analyses.

## Results

### Genotype and allele frequencies of MMP9-1562C/T in CHD patients and controls

**Fig. 1:**
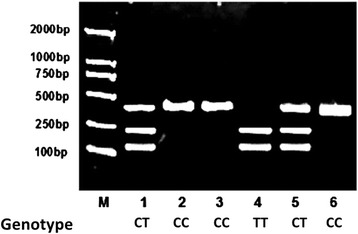
Genotyping of the matrix metalloproteinase 9 rs3918242 by polymerase chain reaction-restriction fragment length polymorphism (PCR-RFLP). Lane 1&5:heterozygote CT;lane 2, 3&6:homozygote CC;lane 4:homozygote TT

We recruited 264 CHD patients who underwent coronary angiography in the fourth clinical hospital of Harbin Medical University during 2013–2014 (112 females, 152 males; age range 55–78 years). 186 control subjects were age and sex-matched healthy subjects who had a routine health check at the medical examination center of Harbin Medical University (90 females, 96 males; age range 52–79 years). Genotyping for MMP9 rs3918242 polymorphism were performed in all the CHD subjects using the genomic DNA extracted from peripheral blood leukocytes (Fig. 1). The genotype distributions in patients with CHD and controls were present in Hardy-Weinberg equilibrium. As shown in Table 1, the genotype and allele frequencies of MMP9 rs3918242 polymorphism were similar between the control subjects and patients with CHD (*P* > 0.05).

**Table 1:** Genotype and allele frequencies of MMP9 rs3918242 in CHD and control subjects

Group	n	Genotype			Allele	
		CC (%)	CT (%)	TT (%)	C (%)	T (%)
Controls	186	151 (81.2)	31 (16.7)	4 (2.1)	333 (89.5)	39 (10.5)
CHD	264	188 (71.3)	69 (26.0)	7 (2.7)	445 (84.3)	83 (15.7)

*CHD* coronary heart disease, *MMP-9* matrix metalloproteinase-9

### Baseline clinical characteristics of patients with CHD and control subjects

Table 2 summarizes the clinical characteristics of the CHD patients and control subjects. All patients had at least one risk factor for coronary artery disease, including smoking, alcohol abuse and lack of exercise. Coronary heart disease was strongly associated with smoking (CHD patients compared with controls: *P* <0.05). At baseline, patients with CHD had significantly higher levels of serum TG, TC, LDL-C, VLDL-C, MMP-9, TNF-α and IL-10 and lower level of HDL-C than control subjects (*P* <0.05).

**Table 2:** Baseline characteristics of patients with CHD and control subjects

	Controls (*n* = 186)	CHD group (*n* = 264)
Sex (M:F)	96: 90	152: 112
Age (years)	58 ± 10.72	59 ± 11.67
Waist (cm)	86.25 ± 6.03	88.11 ± 5.97
Height (cm)	171.28 ± 5.39	169.15 ± 6.62
Weight (kg)	70.04 ± 7.91	71.24 ± 7.35
Body mass index (kg/m^2^)	23.98 ± 2.93	24.73 ± 2.86
Systolic blood pressure (mmHg)	123 ± 19	125 ± 21
Diastolic blood pressure (mmHg)	60 ± 17	65 ± 18
Heart rate	64 ± 15	67 ± 16
Fasting glucose (mmol/l)	4.35 ± 1.02	4.62 ± 1.31
Smoking	45 (24.19%)	109 (41.23%)*
Alcohol abuse	26 (14.05%)	43 (16.27%)

Data are means ± SD. *P <0.01 compared with control group. *CHD* coronary heart disease

### Effects of simvastatin on plasma lipids and MMP-9,TNF-α and IL-10

After 12-weeks simvastatin treatment in combination with the low-fat diet, serum TG, TC, LDL-C and VLDL-C levels were significantly decreased and the HDL-C level was increased in patients with CHD. Furthermore, plasma MMP9, TNF-α and IL-10 were also markedly reduced in CHD patients (Table 3).

**Table 3:** Plasma lipid profile and inflammatory mediators before and after simvastatin treatment in patients with CHD and control subjects

	Controls (*n* = 186)	CHD group (*n* = 264)	
		Before treatment	After treatment
TG (mmol/l)	1.15 ± 0.35	1.58 ± 0.58*	1.14 ± 0.71^§^
TC (mmol/l)	4.11 ± 0.37	5.14 ± 0.49*	4.35 ± 0.53^§^
HDL-C (mmol/L)	1.37 ± 0.31	1.10 ± 0.25*	1.29 ± 0.27^§^
LDL-C (mmol/L)	2.00 ± 0.51	3.1 ± 0.27*	2.32 ± 0.30^§^
VLDL-C (mmol/L)	0.75 ± 0.21	1.03 ± 0.24*	0.78 ± 0.16^§^
MMP-9 (ng/ml)	40.78 ± 9.72	78.17 ± 21.43*	60.32 ± 20.30^§^
TNF-α (pg/ml)	81.23 ± 39.07	136.95 ± 52.41*	102.37 ± 47.19^§^
IL-10 (pg/ml)	32.18 ± 12.15	98.65 ± 34.79*	50.31 ± 24.28^§^

Values are expressed as mean ± SD. Comparison between controls and *CHD* subjects were by unpaired student *T*-test; comparison between before and after treatment of statin were by paired student *T*-test. *P <0.0001 compared with control group; ^§^ *P* <0.0001 compared with before treatment. *CHD* coronary heart disease, *MMP9* matrix metalloproteinase 9, *IL-10* interleukin-10, *TNF-α* TNF-alpha, *TC* total cholesterol, *TG* triglyceride, *HDL-C* high density lipoprotein cholesterol, *LDL-C* low density lipoprotein cholesterol, *VLDL-C* very low density lipoprotein cholesterol

### Influence of the MMP9 rs3918242 polymorphism on plasma levels of lipid and inflammatory mediators

**Fig. 2:**
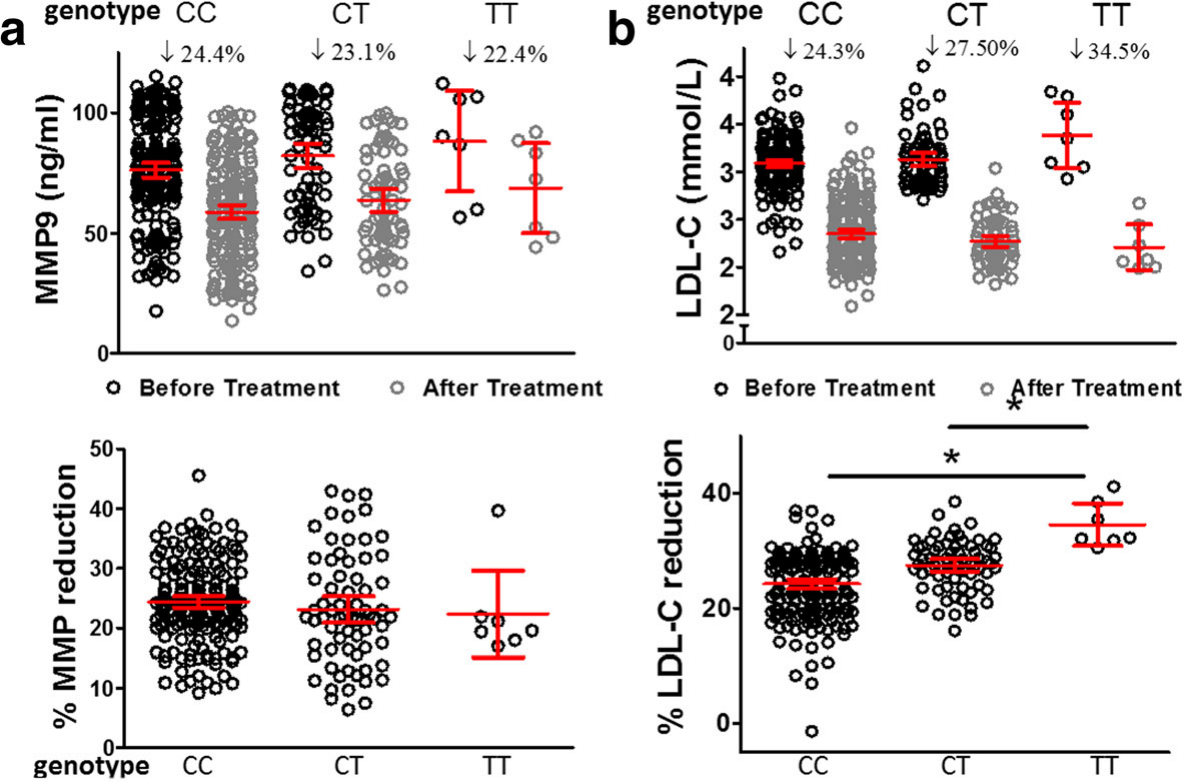
Concentrations of MMP9 and LDL-C in the blood, and percentage of concentration reduction in CHD patients and control subjects before and after simvastatin treatment. **a** Plasma MMP9 concentration in CHD patients before and after simvastatin treatment (*the left graph*), and the percentage of plasma MMP9 concentration reduction in CHD. **b** Serum concentration of LDL-C in CHD patients before and after simvastatin treatment (*the left graph*), and the percentage of serum LDL-C concentration reduction in CHD. Error Bars represent 95% confident interval (95% CI). *n* = 188 subjects with CC, 69 subjects with CT and 7 subjects with TT genotype. *P <0.05, by One-Way ANOVA followed with Bonferroni pairwise test

Given that MMP9 rs3918242 polymorphism has been suggested to regulate MMP expression [17, 18], we first determined the effect of the rs3918242 polymorphism on plasma MMP9 level. The average level of MMP9 in subjects with TT genotype was similar to subjects carrying CT and CC genotypes in this cohort (Table 4 and Fig. 2A). Serum TG and LDL-C levels of patients carrying TT genotype were significantly increased compared to patients carrying CC genotype (*P* <0.05), while HDL-C, VLDL-C, TC, TNF-α and IL-10 levels were comparable in three groups before statin treatment (Table 4 and Fig. 2B). Statin treatment significantly elevated plasma HDL-C, and reduced the levels of LDL-C, TG, TC and VLDL-C in three groups of CHD patients. Notably, the reduction of LDL-C in patients carrying TT genotype was more robust than the LDL-C reduction in patients carrying CC genotype after statin treatment (34.53% vs. 24.23%, *P* <0.05). Interestingly, the reduction of TG, TC, VLDL-C, MMP-9, TNF-α and IL-10 were similar in three groups of patients (Table 4). These findings revealed a novel link of MMP9 rs3918242 TT genotype to the increased serum LDL-C and TG at baseline, and more robust LDL-C-lowering response to statin treatment compared to CHD patients carrying CC genotype.

**Table 4:** Effect of simvastatin in CHD patients with MMP-9 rs3918242 polymorphism

	week	CC (*n* = 188)		CT (*n* = 69)		TT (*n* = 7)	
		Mean ± SD	% Change	Mean ± SD	% Change	Mean ± SD	% Change
TG (mmol/L)	0	1.52 ± 0.19	28.13 ± 3.	1.73 ± 0.25	28.19 ± 3.0	1.87 ± 0.27*	28.12 ± 3.22
	12	1.11 ± 0.21	29	1.21 ± 0.18	7	1.39 ± 0.23	
TC (mmol/L)	0	5.15 ± 0.61	18.24 ± 2.	5.11 ± 0.59	18.59 ± 3.0	5.12 ± 0.51	18.71 ± 3.26
	12	4.34 ± 0.47	67	4.36 ± 0.49	6	4.37 ± 0.56	
HDL-C (mmol/L)	0	1.10 ± 0.19	15.23 ± 2.	1.09 ± 0.18	14.87 ± 2.6	1.13 ± 0.21	15.61 ± 2.42
	12	1.29 ± 0.23	54	1.29 ± 0.22	1	1.29 ± 0.22	
LDL-C (mmol/L)	0	3.09 ± 0.26	24.23 ± 5.	3.13 ± 0.28	27.50 ± 4.4	3.38 ± 0.37*	34.53 ± 4.0
	12	2.35 ± 0.32	78	2.27 ± 0.24	4	2.10 ± 0.26*	0*
VLDL-C (mmol/L)	0	1.04 ± 0.16	34.16 ± 4.	1.01 ± 0.19	34.89 ± 4.5	1.08 ± 0.23	35.14 ± 4.6
	12	0.79 ± 0.09	31	0.75 ± 0.11	7	0.81 ± 0.13	9
MMP-9 (ng/ml)	0	76.28 ± 21.26	24.38 ± 6.59	82.28 ± 21.26	23.10 ± 9.27	88.28 ± 22.47	22.37 ± 7.82
	12	58.75 ± 20.21		63.75 ± 20.21		68.75 ± 20.21	
TNF-α (pg/ml)	0	134.73 ± 51.38	26.48 ± 4.11	137.08 ± 55.63	25.07 ± 5.12	135.98 ± 60.12	24.46 ± 2.99
	12	100.26 ± 49.34		106.65 ± 50.04		99.65 ± 43.87	
IL-10 (pg/ml)	0	100.13 ± 36.17	47.33 ± 9.28	95.08 ± 34.79	52.04 ± 11.62	103.35 ± 40.14	48.27 ± 10.56
	12	51.28 ± 26.21		47.29 ± 29.06		53.91 ± 22.38	

Values are expressed as mean ± SD. *P <0.0001 compared with the CC genotype by one-way ANOVA followed with Bonferroni post-hoc test for pair-wise comparison. ^§^ *P* <0.0001 compared with before treatment by paired student’s *t*-Test. *HD* coronary heart disease, *MMP-9* matrix metalloproteinase-9, *TC* total cholesterol, *TG* triglyceride, *HDL-C* high density lipoprotein cholesterol, *LDL-C* low density lipoprotein cholesterol, *VLDL-C* very low density lipoprotein cholesterol

## Discussion

In this study, we showed that CAD patient carrying MMP9 rs3918242 TT genotype had significantly increased plasma TG and LDL-C levels than patients carrying CC genotype, and their LDL-C lowering response to simvastatin treatment were more robust than patients with CC genotype. These findings revealed a novel link of the MMP9 SNP polymorphism to LDL-C lowering response to simvastatin treatment in CHD patients.

Studies have consistently supported the correlation of MMP-9 rs3918242 polymorphism correlated with increased susceptibility to a variety of diseases [19–21], including coronary artery diseases [22]. The previous study also revealed that MMP-9 rs3918242 polymorphism was associated with the susceptibility to acute coronary syndrome (ACS) in the Uygur population of China [23]. In the present study, we examined the effects of the MMP9 rs3918242 polymorphism on plasma MMP-9 level before and after simvastatin treatment in Chinese CHD patients. We found that plasma MMP-9 levels were elevated in patients with CHD compared to control subjects, and simvastatin treatment reduced plasma MMP-9 in CHD patients. However, MMP9 levels in patients with different MMP9 rs3918242 polymorphism were comparable before and after simvastatin treatment. Early studies revealed that the MMP9 rs3918242 CC genotype was responsible for lower activity of MMP-9 and genotypes with the T allele (CT, TT) were responsible for higher activity [18, 24]. The T-alleles’ effect on circulating MMP-9 levels exclusively in obese and lack of such influence in lean people has further been reported [25, 26]. Because the recruited CHD patients in the current cohort were not obese (average BMI = 24.73), our report further confirmed that the T-allele’s effect on circulating MMP9 level were lacking in non-obese people.

Studies in humans and animal models have provided evidences for a significant involvement of in atherosclerotic plaque progression, through degrading extracellular matrix, destabilizing advanced atherosclerotic plaques and facilitating vascular smooth muscle cell migration and proliferation [3, 27, 28]. Emerging evidences suggest that MMP-9 is also involved in lipoprotein modification during progression of atherosclerotic lesions [29]. Higher levels of MMP-9 have been consistently observed to correlate with increasing total cholesterol or LDL-C levels in humans and animal models [30, 31], suggesting a potential link of MMP-9 rs3918242 polymorphism to serum LDL-C. In this study, we showed that CHD patients with MMP-9 rs3918242 genotype had elevated serum TG and LDL-C levels before simvastatin treatment. More important, we showed that patients with TT genotype had significantly more robust LDL-C lowering effect in response to simvastatin treatment than those with CC genotype. Although the association of LDL-C level with MMP9 polymorphism has been reported in other studies [32, 33], this study is the first to demonstrate that MMP9 polymorphism is associated with LDL-C lowering efficacy after simvastatin treatment, and thus provides new evidences on the link of genetic variations to the efficacy of lipid lowering medications.

This study has several limitations. (1) All the CHD patients were given simvastatin in combination with low-fat diet intervention. Therefore, the LDL-C lowering effect could possibly be biased by the diet intervention. Future studies without diet intervention would be helpful to address this problem. (2) The current CHD cohort contains 41.23% current smokers. It was reported that MMP9 level in the blood was significantly higher in current smokers compared to never and former smokers [34]. Hence, it would be important to analyze the serum MMP9 level and LDL-C lipid lowering effect with further stratification for smoking. (3) Patients in this cohort were uniformly treated with the medium dose of simvastatin (20 mg/day) for 12 weeks, and their serums LDL-C was significantly reduced to the level comparable to the control subjects. In this context, we found that patients carrying MMP9 rs3918242 TT genotype had more robust LDL-C-lowering response to statin treatment compared to patients carrying CC genotype. It would be interesting to examine whether the association of genetic variation of MMP9 rs3918242 with simvastatin’s LDL-C lowering response was dose-dependent. We expected improved LDL-C-lowering response of MMP9 rs3918242 T-allele to simvastatin even at lower dose.

## Conclusions

In conclusion, MMP9 rs3918242 T-allele is associated with elevated plasma TG, increased LDL-C and improved LDL-C-lowering response to simvastatin treatment in Chinese patients with CHD. Further studies with larger sample sizes are required to confirm these findings and to explore the underlying mechanisms.

## Abbreviations

CHD: Coronary heart disease
HDL-C: High-density lipoprotein cholesterol
HMG-CoA: 3-hydroxy-3-methylglutaryl-coenzyme A
LDL-C: Low-density lipoprotein cholesterol
MI: Myocardial infarction
MMP: Matrix metalloproteinase
TC: Total cholesterol
TG: Triglyceride
